# Simulations reveal that different responses to cell crowding determine the expansion of *p53* and *Notch* mutant clones in squamous epithelia

**DOI:** 10.1098/rsif.2021.0607

**Published:** 2021-10-13

**Authors:** Vasiliki Kostiou, Michael W. J. Hall, Philip H. Jones, Benjamin A. Hall

**Affiliations:** ^1^ Department of medical physics and biomedical engineering, UCL, Gower Street, London WC1E 6BT, UK; ^2^ MRC Cancer Unit, University of Cambridge, Hutchison-MRC Research Centre, Box 197, Cambridge Biomedical Campus, Cambridge CB2 0XZ, UK; ^3^ Wellcome Trust Sanger Institute, Hinxton CB10 1SA, UK

**Keywords:** cell competition, clonal evolution, somatic mutation, epithelial dynamics, spatial modelling

## Abstract

During ageing, normal epithelial tissues progressively accumulate clones carrying mutations that increase mutant cell fitness above that of wild-type cells. Such mutants spread widely through the tissues, yet despite this cellular homeostasis and functional integrity of the epithelia are maintained. Two of the genes most commonly mutated in human skin and oesophagus are *p53* and *Notch1*, both of which are also recurrently mutated in cancers of these tissues. From observations taken in human and mouse epithelia, we find that clones carrying *p53* and *Notch* pathway mutations have different clone dynamics which can be explained by their different responses to local cell crowding. *p53* mutant clone growth in mouse epidermis approximates a logistic curve, but feedbacks responding to local crowding are required to maintain tissue homeostasis. We go on to show that the observed ability of *Notch* pathway mutant cells to displace the wild-type population in the mouse oesophageal epithelium reflects a local density feedback that affects both mutant and wild-type cells equally. We then show how these distinct feedbacks are consistent with the distribution of mutations observed in human datasets and are suggestive of a putative mechanism to constrain these cancer-associated mutants.

## 1. Introduction

Ageing human organs accumulate somatic mutations [1]. This is exemplified by the skin and oesophagus, in which mutant clones replace the majority of the epithelium by old age [2–5]. Several of the mutant genes occur recurrently in cancer, arguing that the tumours originate from the mutant clones in normal-appearing tissues. Examples include *p53*, *NOTCH1* and *NOTCH2*, commonly mutated in both normal tissues and cancers in skin and oesophagus. How tissues are able to carry a high burden of oncogenic mutant clones and yet remain functional has not been resolved.

**Figure 1.**
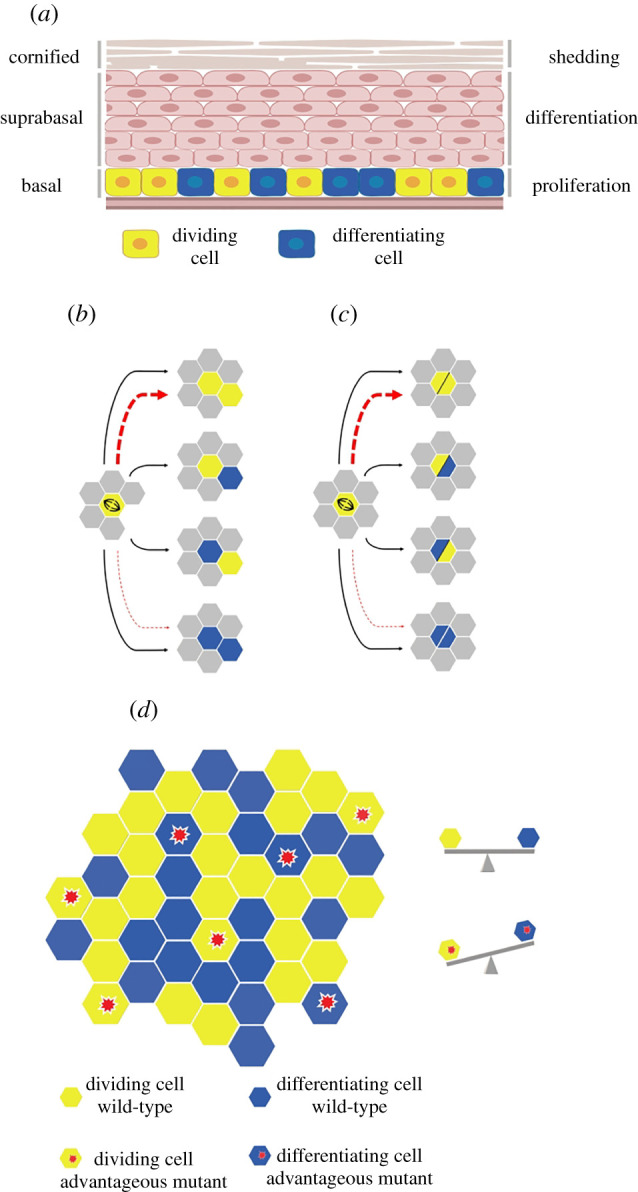
Spatial rules of division and stratification in the simulated tissue. (*a*) The architecture of murine stratified squamous epithelial tissues. Proliferation is restricted to the lowest basal layer. Upon differentiation, basal cells exit the cell cycle and migrate through suprabasal layers, until eventually, they are shed from the tissue. Cell production and loss should be perfectly balanced so that homeostasis and proper tissue function is achieved. Figure was generated using BioRender.com. (*b*,*c*) Schematic representation of the rules of the spatial SP model. Proliferating cells in yellow and differentiating cells in blue. Proliferating cells undergo a division type with balanced division outcome probabilities. In the case of mutant cells, division probabilities are biased, favouring symmetric division, indicated by dashed red lines. A proliferating cell which is about to divide checks its immediate neighbourhood for available space. If a vacant site exists (*b*), one daughter cell occupies the mother cell's space and the second the neighbouring empty space. If there is no empty space in the immediate neighbour (*c*), the two daughters occupy the mother cell's space, thus creating a double-cell occupancy. Double-state cells are released once a neighbouring lattice site becomes available. (*d*) Illustration of the two-dimensional hexagonal lattice representing the epithelial basal layer. Proliferating cells are able to divide, while differentiating cells exit the basal layer and are removed from the simulation. Mutant cells, marked with a red asterisk, have a bias in producing proliferating daughters.

Both skin and oesophagus consist of layers of keratinocytes (figure 1*a*). Proliferation is confined to the deepest, basal cell layer [6,7]. Cell division generates cells that either go on to divide or differentiate, exiting the cell cycle and migrating out of the basal layer into the suprabasal cell layers, eventually reaching the tissue surface from which they are shed (figure 1*b*). Continual cell shedding creates a requirement for constant cell proliferation. Cellular homeostasis requires that on average, each cell division generates one dividing and one differentiating daughter.

The dynamics of dividing cells in mouse skin and oesophagus is described by a simple, non-spatial mathematical model [6–10]. The single progenitor (SP) model proposes that a single, equipotent progenitor cell population maintains the tissue. Progenitor cells divide regularly with an overall division rate *λ*. Divisions give rise to either two progenitor daughters (*AA*), two differentiating daughters (*BB*) or one daughter of each type (*AB*). Division outcome probabilities are balanced, allowing homeostasis to be achieved across the progenitor population (equation (1.1)):

1.1 $$\begin{array}{c} A\overset{\lambda }{\to }\{\begin{array}{cc} AA & r\\ AB & 1-2r\\ BB & r \end{array}\;\\ B\overset{\Gamma }{\to }C\;\\ C\overset{\mu }{\to }\emptyset ,\; \end{array}$$

where *A* represents basal layer progenitor cells, *B* basal cells committed to differentiate and *C* differentiating suprabasal layer cells. Given the fact that *AA* symmetric division leads to clone expansion and *BB* symmetric division tends towards clone extinction, the two symmetric division rates should be equal in order for a steady state in terms of number of cells to be maintained across the progenitor clone population. The probabilities of symmetric and asymmetric divisions are *r* and 1 − 2*r,* respectively, with 0 < *r* < 0.5. Differentiating daughters in the basal layer stratify to the suprabasal layer at rate *Γ* and supra basal cells *C* are shed at rate *μ*. The model captures average cell behaviour in homeostasis and the neutral competition between wild-type cells.

The SP model does not include any consideration of the spatial location of cells. In wild-type epidermis in homeostasis, live imaging studies have also shown that cell division is spatially coupled to the exit of a nearby differentiating cell from the basal layer [11]. Such coupling, together with the uniformity of the proliferative cell compartment in skin and oesophagus, explains the ability of a non-spatial model to describe normal cell dynamics. In transgenic models, mutations of *p53* and a dominant-negative mutant of *Maml1 (DN_Mam1),* which *inhibits the Notch pathway*, have been induced in scattered single cells in the epidermis and oesophagus, respectively [12,13]. The SP model is also able to capture the behaviour of mutant clones at the early stages of expansion by introducing an imbalance in the probabilities of symmetric cell division outcomes (AA and BB). At later stages mutant clones fuse, and only the area of the tissue colonized by mutant cells rather than the cell number in individual clones is measurable. At this stage, however, experimental findings diverge widely from the predictions of the SP model with an imbalance. In both mutant *p53* and *DN_Maml1* mouse models, colonized tissues remain normal in appearance and short-term cell tracking indicates that progenitor cell division outcomes have reverted to balance with an equal probability of AA and BB division outcomes within mutant areas [12,13].

The reversion of mutant cell fate from a proliferative bias towards homeostatic behaviour may also explain how human tissues tolerate a high burden of diverse mutations. The proliferating cell compartments of skin and oesophagus are continuous cell sheets with no barriers to restrict the spread of mutant clones. This allows mutant clones to expand until they collide with adjacent clones, with fittest clones eliminating the less fit. Clones thus compete for the fixed space within the proliferative cell layer, explaining the genetic evidence of mutant selection seen in mouse and human epithelia [2,3,5,14]. In mutagen-treated mice, cells at the edge of clones continue with a bias towards proliferation until they encounter a clone of similar fitness, when the edge cells revert towards homeostasis [14]. The mechanisms that underpin such fitness sensing and ‘neighbour constraint’ are yet to be defined. Candidates include the mechanosensing ion channel PIEZO1 [15,16] and the *Notch, Shh* and *BMP* signalling pathways [17,18].

Here, we use a new computational model of clonal competition (figure 1*b*–*d*), to study the clonal dynamics of mutant *p53* and *DN_Maml1* clones in transgenic mouse epidermis and oesophagus. We find the observed differences in mutant clone dynamics can be explained by different spatial feedback rules, in response to local cell crowding. This model is consistent with the known response of epithelial cells to crowding: increased areal density of keratinocytes promotes their differentiation and exit from the proliferative cell layer [19,20]. However, mutant *p53* and *DN_Maml1* mutations respond differently to crowding influencing the way each mutant spreads through the tissue. We go on to explore the dynamics between simulated *p53* and *DN_Maml1* mutant clones competing within the same tissue, showing that the *Notch* pathway mutations can be expected to reliably outcompete *p53* mutant clones over long periods of time, consistent with human data.

## 2. Results

### 2.1. The spread of *p53* and *DN_Maml1* carrying clones cannot be reconciled with existing models

**Figure 2.**
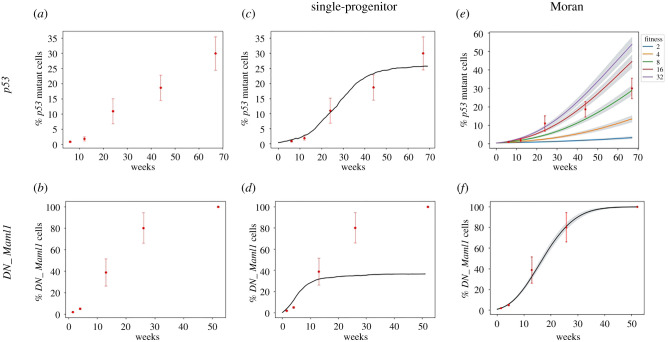
*p53* and *DN_Maml1* mutations in mouse epithelia have been shown to have distinct tissue colonization potential that cannot be reproduced by existing models. (*a*,*b*) *DN_Maml1* mutants fully colonize the tissue, whereas *p53* do so only partially. Figures show the tissue takeover percentage of *p53* and *DN_Maml1* mutations, as measured in [12,13]. Red dots with error bars correspond to observed mean values with s.e.m. (*c*,*d*) The SP model cannot capture the observed complete tissue takeover by *DN_Maml1* mutated cells, and it is more appropriate to reproduce the phenotypic plasticity of the *p53* mutations. This is implemented as a rule-based model to limit growth to the tissue size. Simulations of the rule-based model were performed using Bio-PEPA, a framework for modelling biochemical networks [21]. (*e*,*f*) Moran-style two-dimensional model. The Moran process is unable to reproduce tissue takeover, either overestimating or underestimating takeover depending on the specific choice of parameters. In contrast, it appears consistent with *DN_Maml1* growth.

The study of *p53* and *DN_Maml1* clone dynamics in mouse epithelia has indicated that the mutant progenitor cells have a competitive advantage over their wild-type counterparts, causing the mutant population to expand super-linearly [12,13] (electronic supplementary material, figure S1). This is enabled by an imbalance (*δ*) in the SP model, where divisions resulting in pairs of progenitor cells become more likely than divisions resulting in differentiated daughters, leading to the exponential growth of clones. However, the two mutant types differ in their ability to colonize epithelia [12,13] (figure 2*a*,*b*). Murai *et al*. [12] studied the fate of epidermal epithelial cells carrying a heterozygous p53 gain-of-function mutation *p53^R245W^*, the mouse equivalent human *p53^R248W^* which is frequently detected in normal human epidermis. By inducing a *p53^R245W^* mutation in scattered single epidermal progenitor cells in transgenic mice, the growth dynamics of mutant clones in a background of their wild-type counterparts was tracked. The average *p53* mutant clone size increased super-linearly up to 24 weeks post-induction indicating their competitive advantage over wild-type cells. After this period of rapid mutant clone growth, the expansion rate of *p53* mutant cells slowed considerably so that 30% of the basal layer was colonized by 15 months after induction of mutant expression (figure 2*a*). The rate of mutant cell division did not alter when the rate of clonal expansion slowed but a small (*ca* 10%) increase in basal layer cell areal density was observed. Together, these findings hint that *p53* mutant progenitor cells adjust their division outcomes in response to alterations in their local cellular environment.

Alcolea *et al*. [13] studied the effects of *Notch* pathway inhibition in mouse oesophageal epithelial cells. To do this, they expressed a dominant-negative form of Mastermind-like 1 protein (*DN_Maml1*) that binds and sequesters the intracellular domain of the activated Notch receptor, preventing it from activating transcription of Notch target genes. The mutant Maml1 protein is fused to green fluorescent protein enabling the mutant cells to be visualized and the size of mutant clones quantitified. *DN_Maml1* was expressed in scattered single cells in the esophageal epithelium and the size distribution of cohorts of the resulting clones was measured for the next two weeks, after which the rapidly expanding clones fused together. Subsequently, the area of mutant epithelium was quantified out to 1-year post-induction (figure 2*b*; electronic supplementary material, figure S1b). The growth rate of *DN_Maml1* mutant epithelium slowed beyond three months, but eventually the entire epithelium was colonized. Once tissue take over was completed, the imbalance in mutant fate reverted towards normal and a new homeostatic state with a faster cell turnover was established.

In seeking to further investigate the different observed behaviours of *p53* and *DN_Maml1* mutants, we performed simulations of the growth of each mutant using a spatial Moran model and a rule-based model with a carrying capacity. In the Moran model, each cell had a pre-defined fitness, and at each step of the simulation, a cell had an opportunity to replace its neighbour based on their relative fitness. In the rule-based model, cell populations were updated following the SP paradigm with a maximum population size. Interestingly, neither of these modelling approaches was able to capture the growth dynamics of both *p53* and *DN_Maml1* mutant populations. The rule-based model was able to recapitulate the tissue colonization by *p53* mutant cells (figure 2*c*), but not *DN_Maml1* growth (figure 2*d*). Conversely, the Moran process was unable to reproduce *p53* growth with a single fitness (figure 2*e*) but it accurately recapitulated the observed tissue takeover of *DN_Maml1* mutants (figure 2*f*).

### 2.2. The distinct growth behaviour of *p53* and *DN_Maml1* mutations can be attributed to their different sensitivity to crowding

Having shown that neither of the models we tested was able to reproduce both *p53* and *DN_Maml1* growth we sought to recapitulate the behaviour of both mutants with a common mechanism. The previously tested modelling approaches disregarded the local spatial interactions between cells in the tissue. Nevertheless, there is growing evidence that spatio-temporal regulation of cell dynamics has a role in both tissue maintenance and disease [22–24]. Spatial competition plays an important role in a wide variety of different scenarios, altering growth curves in ways that reflect both tissue structure and experimental methods [25–27]. The finite size of the basal layer of the tissue constrains clone growth, and the topology of the cells restrains clonal expansion to the periphery of the clone. These features potentially alter growth patterns and undermine the validity of the model. In the SP model specifically, we might expect that average clone size grows initially linearly, but becomes sublinear as clones grow large and only can expand through competition with neighbouring clones at the periphery. Thus, to explore how space changes growth and how it affects the ability of mutant cells to spread within the tissue we developed a spatial SP model (figure 1*b*–*d*) (see Methods for details).

We initially sought to confirm that the SP model continues to predict experimental data when spatial competition is explicitly included, starting from a wild-type tissue. The spatial SP model was initially developed without accounting for any kind of spatial feedbacks that could influence cell fate, similar to the non-spatial model. The simulation outputs revealed that the spatial constraints imposed by the lattice do not cause the model to deviate from the experimental data taken from mouse oesophagus. Specifically, using input parameter values inferred from mouse oesophageal data [7], the spatial model was able to reproduce a set of characteristic quantitative properties of clone size distributions (the hallmarks of a single population of progenitor cells), as described in [6] (electronic supplementary material, figure S2). We further extended this testing to all available datasets and found that the growth patterns of the new model accurately reproduced experimental observations (electronic supplementary material, figure S3).

Having confirmed that neutral systems continue to behave as expected, we moved onto examining the growth of advantageous mutations. Initial spatial simulations of the mutant *p53* colonization of the epidermis modified a set of wild-type cells to have an imbalance in symmetric cell fates. However, visualization of the tissue showed increased packing of the mutant cells and an increased abundance of progenitor cells, leading to tissue overcrowding. As the population of progenitor cells and double cells grows over time (i.e. two cells occupying the same lattice space, see Methods for details), the overall tissue turnover slows as cells no longer have space to divide (electronic supplementary material, figure S4a). This is particularly notable in the mutated regions which become packed earliest and is contradicted by observations which suggest that the tissue is otherwise morphologically normal. This has practical implications for the model; simulations can finish within the lifetime of the mouse when the tissue becomes packed with double occupancies (electronic supplementary material, figure S4b,c), whereas the tissue is found to have an increase in cell density of only approximately 10% [12].

**Figure 3.**
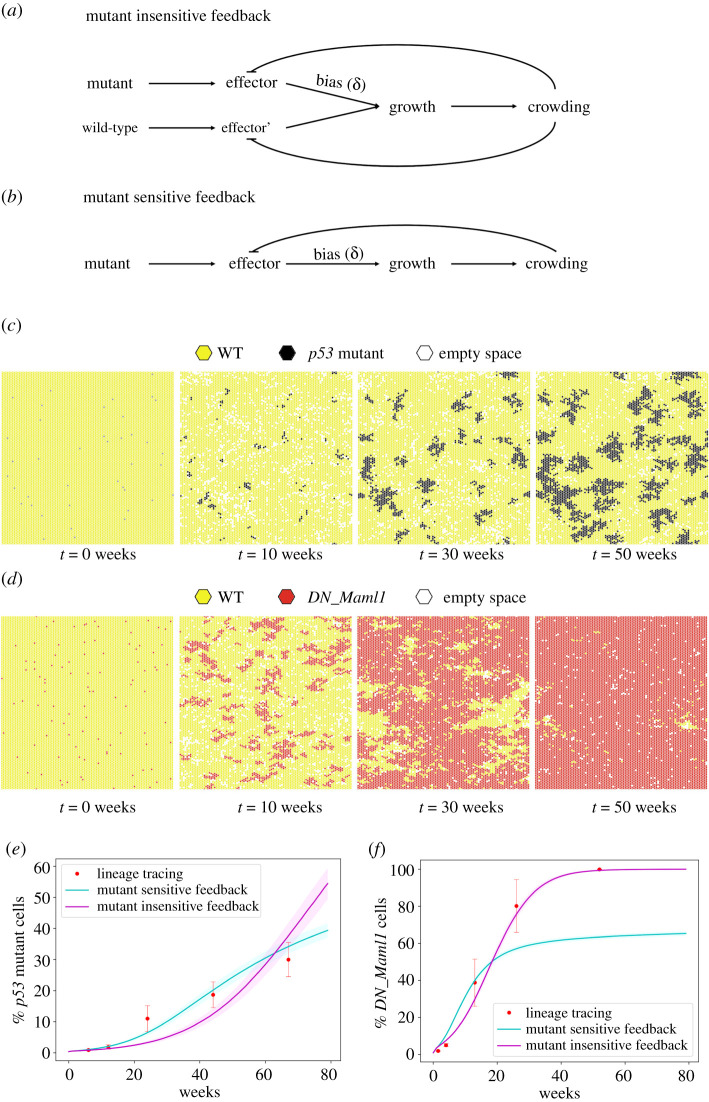
*p53* and *DN_Maml1* growth is explained by two distinct feedback mechanisms. The ‘mutant-insensitive’ (*a*) and ‘mutant-sensitive’ (*b*) feedback mechanisms simulating (*c*) *p53* mutant growth in back epidermis (SP parameters taken from [12]) and (*d*) *DN_Maml1* growth in oesophagus (SP parameters taken from [13]). *p53* mutant growth dynamics is appropriately recapitulated by the mutant-sensitive feedback mechanism whereas *DN_Maml1* by the mutant-insensitive feedback mechanism. (*e*,*f*) Tissue takeover plots show the simulated (solid lines) and observed (red dots) percentage of mutant cells. Data correspond to mean values across 100 simulations. Shaded areas correspond to s.d. Red dots with error bars show observed mean values with s.e.m. Simulation time lapses show *p53* mutant growth with the mutant-sensitive and *DN_Maml1* growth with the mutant-insensitive feedback. Parameters used: *p53*: SP parameters (*λ* = 1.16/week, *r* = 0.06, *Γ* = 3.9) taken from [6], *δ* = 0.95 (from [12]), crowding threshold: seven cells, *DN_Maml1:* SP parameters (wild-type cells: *λ* = 1.9/week, *r* = 0.1, *Γ* = 3.5, mutant cells: *λ* = 6/week, *r* = 0.055, *Γ* = 0.8) taken from [13], *δ* = 1 (from [13]), crowding threshold: six cells, *δ*^′^ = 1.

To address these issues, we extended the model to explicitly enable feedbacks that limit growth in response to local crowding. Cell density increases in large areas replaced by mutant cells and is coincident with a return to neutrality in experiments [13]. Two classes of feedback were tested based on the subpopulation of cells that respond to crowding. In one class, all cells respond to crowding by promoting differentiation (mutant-insensitive feedback) (figure 3*a*). In the second class, only mutant cells respond to crowding by reducing their propensity to symmetric division giving dividing cells (mutant-sensitive feedback) (figure 3*b*).

Using a fixed division rate *λ*, stratification rate *Γ* and symmetric division probability *r*, as inferred in [12],_,_ both approaches were tested at a range of crowding thresholds and induction levels. The mutant-insensitive feedback mechanism enhanced tissue takeover, and no longer matched tissue takeover by the end of the mouse lifetime. By contrast**,** the mutant-sensitive feedback followed the growth curves correctly and was broadly robust to the specific threshold selected (figure 3*c*,*e*). Furthermore, simulations no longer finished in the mouse lifetime and tissue turnover remained roughly constant suggesting that the growth advantage of the mutant *p53* cells is sensitive to crowding, effectively acting as a negative feedback to the mutant cell population. The mutant-sensitive feedback as a more appropriate mechanism to describe mutant *p53* tissue takeover was further confirmed by the fact that it consistently achieved lower RMSE values compared to the mutant-insensitive feedback under various parameter sets (electronic supplementary material, figure S5).

Having shown that the *p53* mutant cell growth can be described by a simple mutant-sensitive feedback, we revisited and retested *DN_Maml1* transgenic mutations in mouse oesophagus. Testing with both feedback mechanisms, we found that the mutant-insensitive feedback was required to capture the rapid takeover, as it matched the experimental observations on *DN_Maml1* tissue colonization. This is consistent with prior observations that showed, through EdU labelling experiments, that wild-type cells neighbouring mutant *DN_Maml1* clones were induced to differentiate and stratify [13]. Visual examination of the underlying simulations suggested that the difference in growth patterns between the mutant-insensitive and mutant-sensitive mechanisms arises because in the mutant-insensitive feedback, the reduction in *δ* in all cells had the effect of ensuring that cells continued dividing, but maintained the relative advantage of the mutant clone (figure 3*d*,*f*).

### 2.3. Competition

Having shown that both sets of mutations can be understood in terms of the interactions of the underlying pathways and the tissue, we sought to assess how simulations with both feedbacks behaved and how the mutations coexist and interact in the tissue. In humans, the prevalence of mutations varies between normal oesophagus and the cancers that derive from it. *p53* mutants are present in the great majority of cancers but are typically present in less than 10% of normal cells [2,3]. This implies that cancers develop from the *p53* mutant population. By contrast, *NOTCH1* mutations are several fold more common in normal oesophagus than in cancers, hinting that the loss of *NOTCH1* function may impede malignant transformation. To explore the interactions of the mutations, we designed a series of different mutational events to consider how the different mutations interact; co-induction, timed inductions and variable levels of relative induction. As mutations that block *NOTCH1* function can be modelled by *DN_Maml1*, we modelled the effect of *NOTCH1* mutations as an increase in the imbalance combined with the measured increase in division rate, in order to examine how the different growth behaviours in the tissue alter the competition.

**Figure 4.**
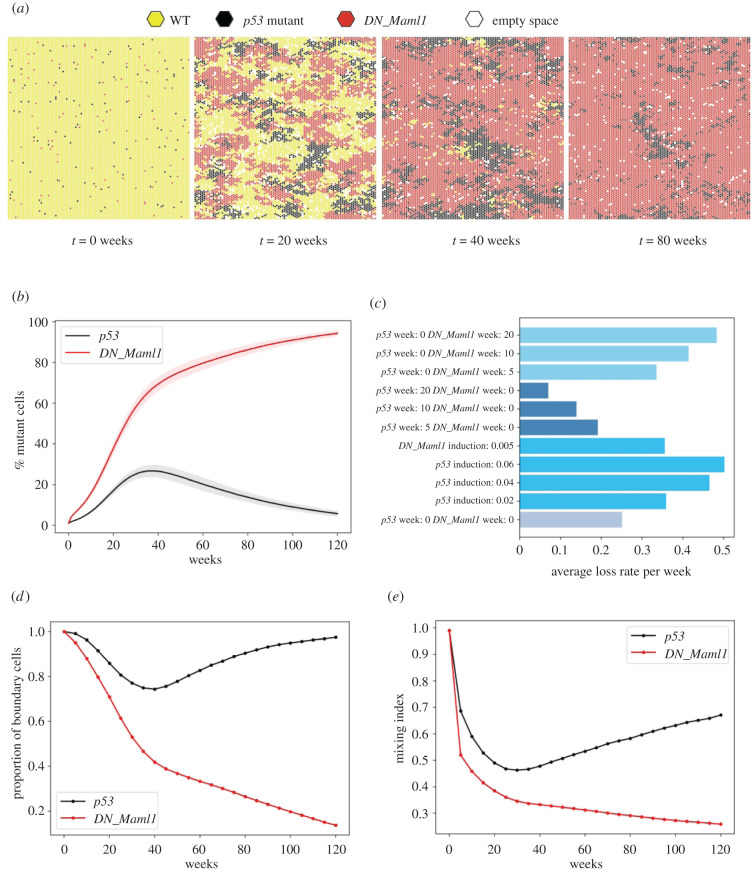
*DN_Maml1* mutations outcompete *p53* mutant cells in competition simulations of oesophagus. (*a*) Typical competition simulation time lapse assuming mutant co-induction and same induction level. (*b*) Tissue takeover percentage of *p53* and *DN_Maml1* mutations, when both are introduced in the tissue. (*c*) Average percentage of *p53* mutant takeover loss rate across all simulated competition scenarios. Calculations measured the difference in tissue takeover between the week when takeover was the highest until the final week of the simulation. (*d*) The proportion of boundary cells in *p53* and *DN_Maml1* mutant cells. (*e*) Proportion of neighbours of a different mutation type or wild-type (cell mixing index) as computed for *p53* and *DN_Maml1* mutations. The cell mixing index was calculated for each mutant cell and the values were averaged over each mutation. Data correspond to mean values across 100 simulations. Shaded areas correspond to s.d. Parameters used: *DN_Maml1*: SP parameters taken from [13], *δ* = 1 (from [13]), crowding threshold: six cells, *δ*^′^ = 1, p53: SP parameters taken from [7], *δ* = 0.95 (from [12]), crowding threshold: six cells.

We found that in all scenarios *DN_Maml1* mutant clones eventually took over the tissue, excluding *p53* clones (figure 4*a*,*b*). *p53* clones that had grown for extended periods before *DN_Maml1* mutations were introduced regressed more slowly, as the larger clones were more slowly outcompeted, but ultimately, the constant fitness advantage offered by *DN_Maml1* mutations eventually led to the loss of *p53* clones. However, *p53* favouring competition scenarios, where *p53* mutants were introduced several weeks earlier or at a substantially higher proportion, led to a higher rate of *p53* clone regression (figure 4*c*). We finally explored the impact of the feedbacks alone, by setting the mutant division and stratification rates to be the same. We found that the advantage of *DN_Maml1* mutants was retained in these simulations (electronic supplementary material, figure S6), though there was a narrow parameter window where the competing clones became neutral following takeover (electronic supplementary material, figure S6c).

In seeking to quantitatively describe any distinct spatial properties of the two competing mutant populations, we observed that the majority of *p53* mutant cells were consistently found in boundaries with *DN_Maml1* or wild-type populations, implying that *p53* mutant cells were not able to form coherent groups in space. On the contrary, the proportion of boundary *DN_Maml1* mutant cells progressively decreased and was almost minimized at later time points, as they colonized the entire grid (figure 4*d*). Furthermore, the proportion of *p53* mutants' neighbours belonging to a different type (cell mixing index) was consistently higher, indicating that *p53* mutant cells were more likely to share junctions with different cell types and therefore tended to be more dispersed (figure 4*e*). This is consistent with experimental observations [12].

The distinct shapes of different clones might also be able to explain the behaviour observed across different competition scenarios. When *p53* mutants are introduced in the grid in much higher numbers compared to their *DN_Maml1* competitors, they expand and form aggregates more easily and faster. Regions of tissue containing clones carrying *p53* mutations are highly mixed. As such they more quickly encounter other *p53* clones as they spread, reducing their effective advantage and further slowing their colonization. The subsequent contact with *DN_Maml1* mutant cells which start expanding substantially in the following time points would lead to their loss as *DN_Maml1* mutant population is more aggressive.

## 3. Discussion

Normal, aged human skin and oesphagus are both heavily mutated and homeostatic, with the tissue retaining normal histological structure and continuing to function. This is more remarkable given that a subset of mutations exemplified by p53 and Notch mutants confer a strong competitive advantage to mutant clones. For tissue integrity to be retained, these competitive mutants must revert to the neutral behaviour that characterizes wild-type cells in homeostasis. The mutant cells must sense and respond to local cell density within the proliferating cell layer of the epithelium, which remains constant. Sequencing results in humans and lineage tracing in transgenic mice indicate *Notch* pathway mutants are more effective at colonizing squamous epithelia than *p53* mutants. This difference in clonal expansion is likely to be multifactorial, but our results point to one difference between the mutants being their responses to local cell crowding, likely underpinned by differences in the impact of the mutations on gene regulatory networks.

We subsequently used the spatial model with the distinct two feedback mechanisms to explore how mutant *p53* and *DN_Maml1* compete within the same tissue. In a wild-type background, the *p53* mutant cell population was always outcompeted by *DN_Maml1* population. The winning behaviour of *DN_Maml1* over mutant *p53* cells was consistent across a range of competition scenarios. This argues that Notch pathway mutants may impede cancer development by constraining oncogenic *p53* mutants within epithelia. The distinct responses of *DN_Maml1* and *p53* mutants to local cell crowding may therefore not be accidental. Spatial rules favouring the dominance of *DN_Maml1* over *p53* mutants may have been selected through evolutionary pressure in order to reduce cancer risk in tissues.

Finally, this work further raises the question of how to measure and model mutational spread in tissues. A series of papers have illustrated how the effect of clone expansion may be modelled using a simple imbalance in fate or a fitness advantage, and that this could be related to genetic measures of non-neutrality such as the ratio of protein altering to silent mutations (dN/dS) [12–14,25,28]. The fundamental observation that underpins the modelling presented in this work, however, illustrates that any apparent advantage of an individual clone may not necessarily predict its overall takeover of the tissue. In this context, estimates of selection will represent a compound of both clonal advantage and tissue-level effects. Considering the role of feedback systems in the tissue, and how they work to maintain homeostasis, represents a major challenge for future work.

## 4. Methods

### 4.1. Spatial single progenitor model

A stochastic cellular automaton (CA) was used to implement the SP model in two-dimensional space and explore the collective behaviour of cells in the tissue. A two-dimensional, hexagonal lattice was used to model the basal layer of the epithelium, reflecting the observation that each oesophageal basal cell has six neighbours on average (figure 1*d*). Basal epithelial cells were simulated on a lattice originally containing 10 000 cells, *L* = 100 × 100, corresponding to roughly 1% of the area of adult mouse oesophagus, where periodic boundary conditions were applied. Each simulation was repeated 100 times.

Each site of the grid may be occupied either by one of the two cell types described in the SP model, proliferating cells (*A*) and post-mitotic cells (*B*), or it may remain vacant as a result of a stratification event. Also, a lattice site may be occupied by two cells, indicating a crowded region. A division event can lead to three potential outcomes: two proliferating cells, two differentiating cells or one daughter of each cell type. The neighbourhood in the SP CA model is defined by the six adjacent places. Division and stratification events were considered as two independent processes determined solely by *r*, *λ* and *Γ* parameters. This cell-autonomous approach could lead to cases where a cell division event occurs at a region with no available neighbouring vacant space. In this case, the two daughter cells were placed on the same grid space, indicating an increased cell density area. Analogously, cases where an empty space generated by a recently stratified B cell is not rapidly replaced by a nearby newly born cell might be observed, representing a low cell density area. Considering the above, each lattice site could have one of the following seven potential states: *A*, *B*, *D_AA_*, *D_AB_*, *D_BA_*, *D_BB_* and ‘empty’ (∅︀), where *D_AA_*, *D_AB_*, *D_BA_*, *D_BB_* correspond to double occupancies (figure 1*b*,*c*). Thus, the SP model (equation (1.1)) was extended to explicitly include space as follows:

4.1 $$\begin{array}{c} A\;\text{Ø}\overset{\lambda }{\to }\{\begin{array}{cc} AA & r\\ AB & \frac{1}{2}-r\\ BA & \frac{1}{2}-r\\ BB & r \end{array}\;\\ \;\\ A\;X\overset{\lambda }{\to }\{\begin{array}{cc} D_{AA} & r\\ D_{AB} & \frac{1}{2}-r\\ D_{BA} & \frac{1}{2}-r\\ D_{BB} & r \end{array}\;\\ \;\\ B\overset{\Gamma }{\to }\text{Ø},\; \end{array}$$

where *A* ∅︀ denotes a type *A* cell neighbouring a vacant lattice site and *AX* denotes a type *A* cell neighbouring either a type *A* or type *B* cell, thus indicating that there is no neighbouring empty space. *D_AA_*, *D_AB_*, *D_BA_*, *D_BB_* correspond to double occupancies. The CA model was developed in NetLogo [29]. We used a Markovian stochastic simulation algorithm where the basal layer was simulated as an asynchronous CA. The algorithm included the following steps:

1. Start by defining a grid of *NxN* sites (*N* = 100) with *A* and *B* cells randomly seeded.
2. For every cell on each lattice site, draw a random number from an exponential distribution with mean 1/*λ* or 1/*Γ* to assign time of next event (division or stratification) for *A* and *B* cells, respectively.
3. Select cell with the smallest next event time assigned. Current time is updated to the smallest next event time.
4. If an *A* cell is selected, use a random number from a uniform distribution *U* ∈ (0, 1) to choose the division type to occur by comparing *U* to division probabilities. Assign the division type as a next event for the selected cell. If a *B* cell is selected, assign stratification as a next event for the selected cell.
5. If the next event is division, all neighbouring places are checked for empty space. In the case of an existing neighbouring space, one newborn cell will replace the mother cell and the other will occupy the empty neighbouring space. If there is no empty neighbouring space available, then both will remain at the mother cell's space (creating a ‘double-state’ cell), until a neighbouring space is released. If stratification is the next event, *B* cell stratifies, leaving an empty space, which allows potential neighbouring ‘double-state’ daughters to be released.
6. Repeat steps 3–6 until there are no *A* or *B* cells left, or time threshold is reached.

### 4.2. Spatial single progenitor model of non-neutral growth

The study of *p53* and *DN_Maml1* mutant clone dynamics in mouse epithelia indicated that the mutant progenitor clones are not in homeostasis in a mixed tissue and have a fitness advantage over their wild-type counterparts [12,13]. This has been shown to be achieved by having a bias (*δ*) towards the production of proliferating progeny. Such bias results in a gradual expansion in the proliferating population over time as there are less chances for the mutant clones to be lost by differentiation. Thus, mutant clonal expansion appears to be consistent with a SP model including a cell fate imbalance:

4.2 $$\begin{array}{c} A\text{Ø}\overset{\lambda }{\to }\{\begin{array}{cc} AA & r(1+\delta )\\ AB & 1-2r\\ BB & r(1-\delta ) \end{array}\;\\ B\overset{\Gamma }{\to }\text{Ø},\; \end{array}$$

where *δ* denotes the tilt in cell fate. Therefore, *δ* = 0 corresponds to homeostasis, *δ* = 1 implies the absence of symmetric differentiation, leading to persistence and *δ* = −1 implies the absence of symmetric division, leading to extrusion.

To simulate mutant clonal dynamics in two-dimensional space, we used the spatial SP model including a fate imbalance, considering both wild-type and mutant epithelial cells. The choice of the grid architecture, the neighbourhood, the number of states and spatial rules of the CA model were implemented as described in the homeostatic spatial SP model. However, a new mutation-status property was introduced to distinguish mutant cells from wild-type cells. In the case of *p53* cells, mutants have the exact same properties as the wild-type cells and the only thing that distinguishes the two cell populations is that *p53* mutant cells have an innate bias towards the production of proliferating cells (*δ*) (figure 1*b*,*c*). In the case of *DN_Maml1* mutant cells, mutants will also have distinct *λ*, *Γ* and *r* values, as inferred by [13]. Considering the above, the spatial SP model, initially described in equation (4.1) was modified as follows, in order to accommodate the mutant cell population:

4.3 $$\begin{array}{c} A\;\text{Ø}\overset{\lambda }{\to }\{\begin{array}{cc} AA & r(1+\delta )\\ AB & \frac{1}{2}-r\\ BA & \frac{1}{2}-r\\ BB & r(1-\delta ) \end{array}\;\\ \;\\ A\;X\overset{\lambda }{\to }\{\begin{array}{cc} D_{AA} & r(1+\delta )\\ D_{AB} & \frac{1}{2}-r\\ D_{BA} & \frac{1}{2}-r\\ D_{\mathrm{BB}} & r(1-\delta ) \end{array}\;\\ \;\\ B\overset{\Gamma }{\to }\text{Ø},\; \end{array}$$

The simulation steps were modified as follows:

1. Start by defining a grid of *NxN* sites (*N* = 100) with *A* and *B* cells randomly seeded.
2. Insert randomly mutant cells of type *A*.
3. For every cell on each lattice site, draw a random number from an exponential distribution with mean 1/*λ* or 1/*Γ* to assign time of next event (division or stratification) for *A* and *B* cells, respectively.
4. Select cell with the smallest next event time assigned. Current time is updated to the smallest next event time.
5. If an *A* cell is selected, use a random number from a uniform distribution *U* ∈ (0, 1) to choose the division type to occur by comparing *U* to division probabilities. Symmetric division probabilities for mutant cells are biased according to *δ*. Assign the division type as a next event for the selected cell. If a *B* cell is selected, assign stratification as a next event for the selected cell.
6. If the next event is division, all neighbouring spaces are checked for empty space. In the case of an existing neighbouring space, one new daughter cell will replace the mother cell and the other will occupy the empty neighbouring space. If there is no empty neighbouring space available then both will remain at the mother cell's space (creating a ‘double-state’ cell), until a neighbouring space is released. If stratification is the next event, *B* cell stratifies, leaving an empty space, which allows a pair of potential neighbouring ‘double-state’ daughters to be released.
7. Repeat steps 4–7 until there are no *A* or *B* cells left, or time threshold is reached.

To introduce feedbacks in the non-neutral simulations, we followed the same steps and modified the division probabilities. To perform simulations with the mutant-insensitive feedback, an additional fate bias parameter, *δ*^′^, was introduced. The value of *δ*^′^depends on the local cell density, i.e. number of neighbouring cells. A neighbourhood (*n*) consisting of more than a defined number of cells would be considered as ‘crowded’ whereas fewer neighbouring cells than the defined crowding threshold would indicate an underpopulated region (’empty’). In the former case, *δ*^′^ is negative rendering the fate of dividing cells tilted towards symmetric differentiation to release crowding. In the latter case, a positive *δ*^′^ is chosen, favouring symmetric division to fill the empty sites. When the neighbourhood is neither ‘crowded’ nor ‘empty’, this indicates that the local cell density is homeostatic and therefore *δ*^′^ = 0:

4.4 $$\begin{array}{c} A\;\text{Ø}\overset{\lambda }{\to }\{\begin{array}{cc} AA & r(1+\delta +\delta^{\prime })\\ AB & \frac{1}{2}-r\\ BA & \frac{1}{2}-r\\ BB & r(1-\delta -\delta^{\prime }) \end{array}\;\\ \;\\ A\;X\overset{\lambda }{\to }\{\begin{array}{cc} D_{AA} & r(1+\delta +\delta^{\prime })\\ D_{AB} & \frac{1}{2}-r\\ D_{BA} & \frac{1}{2}-r\\ D_{BB} & r(1-\delta -\delta^{\prime }) \end{array}\;\\ \;\\ \delta^{\prime }\to \begin{array}{cc} + & n<\mathrm{cell}\;\mathrm{density}\;\mathrm{cutoff}\\ 0 & n=\mathrm{cell}\;\mathrm{density}\;\mathrm{cutoff}\\ - & n>\mathrm{cell}\;\mathrm{density}\;\mathrm{cutoff} \end{array}\;\\ B\overset{\Gamma }{\to }\text{Ø},\; \end{array}$$

where *δ*^′^ ∈ [0, 1]. As each cell on the grid has six neighbours in normal conditions, i.e. no overcrowding, no gaps, the local cell density crowding cut-off was set to six cells. This reflects the topology of the healthy tissue. Furthermore, as wild-type cells do not have a *δ*, they would solely respond to the cell density bias (*δ*^′^), whereas in the case of mutant cells the cell density bias (*δ*^′^) and their innate fate bias (*δ*) would be counterbalanced (equation (4.4)). To avoid large increases in overall tissue cell density, an additional rule was applied to every cell in the grid that had an overcrowded neighbourhood (*n* > 8 cells). In this case, the probability of that cell undergoing symmetric division was minimized (*AA* = 0, *BB* = 2*r*).

To perform simulations with the mutant-sensitive feedback mechanism, no additional cell density fate bias parameter (*δ*^′^) was used but mutants were set to lose their innate bias towards the production of proliferating progeny, (*δ*), in response to crowding in their local neighbourhood. Thus, the mutant-sensitive feedback model can be described by equation (4.3) which describes a spatial model with fate bias. The value of *δ* parameter is turned off when the local cell density (i.e. number of cells) of a dividing mutant cell's neighbourhood consists of more than a defined crowding threshold, switching the mutant behaviour to balanced mode (*δ* = 0). Moreover, to allow for crowding release, induced by the double-state cells observed in mutant-sensitive feedback simulations, we introduced an additional rule where every new division that gave rise to a double occupancy consisting of at least one differentiating cell (*D_AB_*, *D_BA_*, *D_BB_*) would lead to an instantaneous stratification event, that is, the removal of one *B* cell from the simulation. This additional rule is consistent with previous reports of cell extrusion events to compensate proliferation induced local stress [16,30,31].

To perform mutant competition simulations, both mutant-sensitive and mutant-insensitive feedback rules were introduced in the same simulation. *p53* mutant cells followed the mutant-sensitive feedback while *DN_Maml1* mutants and wild-type cells followed the mutant-insensitive feedback.

## Data accessibility

Netlogo models used in this study are included in a ZIP-compressed archive in electronic supplementary material [32], with a readme file describing their use.
